# Arbuscular mycorrhizal colonization defines root ecological strategies in an extreme arid environment

**DOI:** 10.3389/fpls.2024.1488383

**Published:** 2025-01-15

**Authors:** Cristian A. Delpiano, Rodrigo S. Rios, Claudia E. Barraza-Zepeda, Melissa J. Pozo, Lorgio E. Aguilera, Andrea P. Loayza

**Affiliations:** ^1^ Laboratorio de Ecología del Desierto, Departamento de Biología, Universidad de La Serena, La Serena, Chile; ^2^ Instituto de Ecología y Biodiversidad (IEB), Santiago, Chile; ^3^ Departamento de Biología, Universidad de La Serena, La Serena, Chile

**Keywords:** Coastal Atacama Desert, hyper-aridity, fine roots traits, mycorrhizal symbiosis, root economic space, root phenotypic network, desert shrubs

## Abstract

The symbiosis between mycorrhizae fungi and plant roots is essential for plant establishment in nearly all terrestrial ecosystems. However, the role of mycorrhizal colonization (colM) in shaping root ecological strategies remains poorly understood. Emerging research identifies colM as a key trait influencing the multidimensional covariation of root traits within the Root Economic Space (RES), where a ‘collaboration gradient’ is proposed. At one end of this gradient, species with larger root diameters (RD) rely on colM for resource acquisition through an ‘outsourcing’ strategy, while at the other end, species with finer roots and greater exploration capacity employ a ‘do it yourself’ strategy to acquire resources independently. Although the RES framework has improved our understanding of root strategies, the relationship between colM and root traits in desert ecosystems remains underexplored, particularly in hyper-arid environments, where limited resources can constrain both plant and mycorrhizal survival. In this study, we examine the root ecological strategies of 32 dominant shrub species in Chile’s Coastal Atacama Desert, focusing on the link between specific root traits and colM. We found that larger RD correlated with higher levels of colM, supporting the ‘outsourcing’ strategy within the ‘collaboration gradient’ hypothesis of the RES. Additionally, RD and colM emerged as playing key roles in defining both dimensions of root ecological strategies. Moreover, we identified colM as a central hub trait in the root phenotypic network, underscoring its role in survival strategies under hyper-arid conditions. These findings emphasize the critical importance of colM in modulating plant ecological strategies and highlight the need to further investigate how AM enhances root lifespan and optimizes resource uptake in extreme environments.

## Introduction

The mutualistic relationship between mycorrhizae fungi and plant roots is crucial for plant establishment in almost all terrestrial ecosystems. However, how this symbiosis influences plant ecological strategies, particularly the trade-off between resource allocation for growth and survival, remains poorly understood (McCormack and Iversen, 2019; Freschet et al., 2021b). Research into general patterns of root resource acquisition has revealed that co-variation among root traits parallels co-variation among analogous leaf traits, leading to the concept of a one-dimensional axis of variation known as the ‘root economic spectrum’ (Withington et al., 2006; McCormack et al., 2012; Reich, 2014). On one end of this spectrum, traits favor rapid resource uptake and growth (e.g., roots with high nitrogen content [RNC] and specific root length [SRL]); at the other end, traits promote longevity and slower growth (e.g., roots with large diameters [RD] and high tissue density [RTD]) (Weemstra et al., 2016; Roumet et al., 2016). However, recent findings suggest that root traits may vary along two dimensions (Kong et al., 2014; Kramer-Walter et al., 2016), which may be partly explained by their association with arbuscular mycorrhizae (AM). A recent hypothesis highlights the critical role of AM in explaining the multidimensional nature of root resource acquisition strategies (Bergmann et al., 2020). The relationship between AM and roots provides alternative pathways of resource uptake, allowing plants to adapt to changes in resource availability (Ma et al., 2018; McCormack and Iversen, 2019; Bergmann et al., 2020). Understanding the relationship between roots and AM is thus crucial to unraveling the complex variation in root traits and their influence on plant ecological strategies.

Roots exhibit significant phenotypic variation, enabling efficient uptake of water and nutrients (Kenrick and Crane, 1997; Kramer-Walter et al., 2016; Iversen et al., 2017), which is crucial for plant performance in resource-limited environments. However, root traits alone do not always fully capture a plant’s functional root strategy. For most plants (*ca.* 80%), resource acquisition relies on a partnership with mycorrhizal fungi (Smith and Read, 2008; Brundrett, 2009), particularly with AM. Recognizing the role of AM and incorporating them into analyses of root trait co-variation has deepened our understanding of the phenotypic multidimensionality of root traits (McCormack and Iversen, 2019). This approach has led to identifying two primary axes of trait co-variation, referred to as the ‘Root Economics Space’ (RES) (Bergmann et al., 2020). The first axis relates to resource acquisition strategies and is determined by the co-variation between SRL and RD (Ma et al., 2018; Bergmann et al., 2020), which is strongly influenced by the association with AM. Specifically, greater AM colonization is associated with species with larger RD (Comas et al., 2014; Valverde-Barrantes et al., 2017). Along this ‘collaboration gradient,’ species with fine, low-cost roots (e.g., high SRL and low RTD) can explore and obtain soil resources independently (a ‘do it yourself’ strategy) (Bergmann et al., 2020), while species with thicker roots can adopt an “outsourcing” strategy, relying on mycorrhiza for resource acquisition (Bergmann et al., 2020). The second axis aligns with the fast-slow plant economics spectrum (Reich, 2014). It is determined by the co-variation between RNC and RTD, which reflect metabolic rates and tissue conservation (Eissenstat et al., 2015).

The RES has been demonstrated across species from various biomes, but arid ecosystems remain significantly underrepresented, with data available for only a few species (n=10), accounting for less than 2% of the total species sampled (Bergmann et al., 2020). In these environments, AM are particularly important for plant performance, enhancing resistance to water and nutrient scarcity (Khudairi, 1969; Allen, 2007; Qiao et al., 2019). AM also mediate tolerance to other abiotic stressors in deserts, such as heat and salinity (Rodriguez et al., 2008; Madouh and Quoreshi, 2023), and may play a critical role in survival through protection mechanisms (Freschet et al., 2021b). Notably, mycorrhizal colonization tends to increase with environmental stress (Allen et al., 2003; Kivlin et al., 2013; Aguilera et al., 2016), suggesting that the intensity and integration of AM colonization with root traits could indicate the relevance of mycorrhizal associations in shaping resource acquisition strategies and survival in extreme environments (Freschet et al., 2021b).

To better understand the ecological significance of root strategies, the relationship between root traits and AM colonization can be modeled as a phenotypic network (He et al., 2020). In this network, nodes represent root traits, and edges indicate strong correlations between them. This framework helps identify the most central or interconnected traits (i.e., ‘hub-traits’) and their influence on root strategies (Kleyer et al., 2019; Rao et al., 2023). If AM colonization strongly correlates with multiple root traits and spans more than one dimension of trait co-variation, it likely plays a multifaceted role in root strategies, leading to high network connectivity mediated by AM. Despite the potential insights from this network approach, few studies have explored root trait relationships in this way (see Kleyer et al., 2019; Rao et al., 2023; Ye et al., 2024). Incorporating AM colonization into network analysis could reveal its role in shaping root strategies, particularly in extreme desert environments.

Deserts pose significant challenges to many life forms due to their highly variable environmental conditions. In these environments, high aridity can reduce the diversity and abundance of mycorrhizas (Berdugo et al., 2020; Weber et al., 2019), potentially affecting their symbiotic relationship with plants (Millar and Bennett, 2016). Furthermore, as aridity increases, variation of root traits linked to resource acquisition, such as RNC, tends to increase beyond a certain threshold. This suggests that in extremely arid conditions, plants might benefit from adopting strategies that enable rapid resource uptake during brief periods of availability (Carvajal et al., 2019; de la Riva et al., 2021). While plants in arid environments display a range of root strategies to thrive in these challenging conditions, it remains unclear whether these include a ‘collaboration axis’ with mycorrhizal fungi. Expanding predictions of the RES (*sensu* Bergmann et al., 2020) to encompass a broader range of conditions requires studies that consider root trait variation alongside quantitative data on AM in arid ecosystems.

The Coastal Atacama Desert (CAD) in Chile, recognized as the world’s driest desert, is a hyper-arid environment along most of its extent. In this environment, where prolonged droughts can lead to the complete loss of aboveground plant organs, long-lived woody shrubs thrive despite severe water scarcity. Previous studies have shown that these shrubs possess diverse root systems with high and variable root trait covariation along water and nutrient gradients (Squeo et al., 1999; Morales et al., 2014). However, this variation does not always align with the predictions of the RES (Carvajal et al., 2019; Delpiano et al., 2020). The functional role of AM in the CAD remains largely unexplored. Despite this, AM colonization has been documented even in the driest regions of this ecosystem and across various plant families, including those traditionally considered non-mycorrhizal (Dhillion et al., 1995; Aguilera et al., 1998). Although direct evidence linking root functional traits to AM colonization is lacking, it is expected that AM to play a key role in determining the survival and growth strategies of plants in this extreme environment.

In this study, we explore below-ground ecological strategies under the extreme aridity conditions of the CAD. We sampled absorptive fine roots from 32 dominant shrub species to examine root trait co-variation and their relationship with mycorrhizal fungi. We hypothesized that variation in fine root traits at the species level is linked to mycorrhizal colonization, supporting the existence of a ‘collaboration gradient’. We predicted that an increase in fine root diameter in shrubs would correlate with increased mycorrhizal colonization. Additionally, mycorrhizae colonization would be highly interconnected and central in the root trait network (i.e., a hub-node), underscoring the vital role of mycorrhizal associations in shaping root ecological strategies in arid environments.

## Materials and methods

### Study sites and species sampling

We conducted this study across six sites along the CAD (listed from north to south): Pan de Azúcar (PA), Quebrada El León (QL), Llanos de Challe (LLA), Chañaral de Aceituno (CHA), Cuesta Porotitos (PO), and Fray Jorge (FJ). Mean annual precipitation at these sites ranges from 4 to 147 mm, while mean annual temperature remain relatively stable between 13.6 and 22.9°C (**Supplementary Table S1**). Most of the rainfall is concentrated in a few pulses during the winter months (May through September), followed by prolonged droughts lasting up to 10 months at the wettest sites and several years at the driest ones (Carvajal et al., 2019). According to De Martonne’s aridity index (DEMAI) (De Martonne, 1926), all sites, except for FJ (classified as arid), fall into the hyper‐arid category (**Supplementary Table S1**). We measured traits from species that account for *ca*. 90% of the total species abundance at each site, covering 32 shrub native or endemic species to Chile. These species span nine plant orders, 12 families, and 25 genera (**Supplementary Table S2**).

### Functional root traits

During the peak of the aboveground vegetative growth period, from August to November 2022, we collected samples from at least five individuals of each species across the six study sites. Root sampling was conducted by excavating at a depth of 10 to 20 cm beneath the canopy of each plant. We collected fine root samples (< 2 mm diameter, typically including first and second-order roots for most species), sealed them in bags, and stored them in a cooler before transporting them for processing within eight hours (Freschet et al., 2021a). In the laboratory, we first measured fresh weight of the roots (FW, g). Then, we obtained digital images of each sample using a high-resolution scanner (800 dpi resolution, EPSON V850 Pro). We processed these images with the WINRHIZO Basic software (v.2009c; Regent Instruments, Quebec, Canada) to calculate root traits such as mean diameter (RD, mm), volume (V, cm^3^), surface (S, cm^2^) and total length (L, m). After scanning, roots were dried at 60°C for 72 hours to obtain their dry weight (DW, g). From these measurements, we calculated specific root length (SRL, m gr^-1^) as L/DW, root tissue density (RTD, g cm^-3^) calculated as DW/V, specific root area (SRA, cm^2^ gr^-1^) as S/DW), and root dry matter content (RDMC, mg gr^-1^) as DW (mg)/FW). We also quantified root carbon content (RCC, %), root nitrogen content (RNC, %), and root carbon-nitrogen ratio (RC:N) at the Laboratory for Biogeochemistry and Applied Stable Isotopes (LABASI) at Pontificia Universidad Católica de Chile in Santiago, using a Thermo Delta V Advantage IRMS coupled with a Flash 2000 Elemental Analyzer.

### Mycorrhizal root colonization

To estimate the extent of arbuscular mycorrhizae (AM) fungi colonization, we collected fresh fine roots (1-2 mm diameter) from the same plants used for functional trait measurements. We sampled 10 to 20 root segments from each plant, each 1-2 cm long, and preserved them in a Formalin-Acetic-Alcohol fixing solution (FAA). In the laboratory, roots were submerged in 10% KOH at 90 °C, then washed with distilled water and stained with Trypan blue in lactoglycerol, following a modification of the protocol by Phillips and Hayman (1970). This staining process makes AM fungi visible under a microscope. To determine the colonization percentage (colM), we examined ten haphazardly selected root segments from each sample under a microscope (Nikon Eclipse E200, 400× magnification). We quantified AM fungi colonization by assessing each root segment for the presence of arbuscules, vesicles, and hyphae. Root segments with visible AM structures were assigned a 10% colonization, while those without were given 0%. The overall percentage of colonization was then calculated based on these observations.

### Phylogenetic tree reconstruction

To account for the influence of evolutionary history on the variation of root functional traits across species, we constructed a species-level phylogeny for all 32 species in our study. The phylogenetic tree was based on the comprehensive angiosperm phylogeny of Smith and Brown (2018), which is a dated seed plant phylogeny derived from a hierarchical clustering analysis of publicly available molecular data across major Spermatophyta clades, and integrated with data from the Open Tree of Life project. We extracted the phylogenetic information using the get_tree R-function from the *rtrees* package (Li, 2023). To resolve two polytomies in the tree, we applied the multi2di function from the *ape* package (Paradis and Schliep, 2019). The final tree was used to assess the strength of the phylogenetic signal of all root traits and for all subsequent statistical analyses.

### Root trait covariation and mycorrhizal colonization

To explore patterns of root trait covariation and their relationship with mycorrhizal fungi, we conducted a phylogenetic principal component analysis (pPCA) using the *phytools* package (Revell, 2024). Prior to analysis, all traits were log-transformed to normalize the data, except for colM, which was scaled to a 0-1 range and then transformed using the arcsine square root (Bergmann et al., 2020). We used the first two pPCA axes as they captured a substantial proportion of trait variation. The scores from these axes served as proxies for trait covariation, and a RES representing gradients of trait variation across shrub species. To further account for phylogenetic relationships among species, we fitted each trait to colM using a phylogenetic generalized least square model using the pgls R-function in the R package *caper* (Bergmann et al., 2020). Each model included a phylogenetic covariance matrix derived from our assembled tree. This matrix adjusted for the non-independence of traits due to shared phylogenetic history, integrating phylogenetic effects into the residual error structure of our models.

### Trait phylogenetic signal

We estimated the phylogenetic signal for each trait to assess whether closely related species exhibit greater similarity in root traits than expected by chance. We used Blomberg’s K (Blomberg et al., 2003) and Pagel’s λ (1999) metrics to quantify phylogenetic signals in quantitative traits. Blomberg’s K compares the observed trait variation among species to what would be expected under a Brownian motion model of evolution. A K-value of 1 indicates that trait variation follows the Brownian motion model. K-values >1 and <1 imply that close relatives are more similar and less similar, respectively, than expected. If K is not significantly different from zero, it indicates a lack of phylogenetic signal for that trait (Blomberg et al., 2003). Pagel’s λ is a phylogenetic transformation that optimizes the likelihood of observed data under a Brownian motion model. A λ value of 1 indicates that trait evolution aligns with Brownian motion, considering the variance-covariance in trait changes over the phylogeny’s branch lengths. Values between 0 and 1 indicate a weaker phylogenetic signal than expected under Brownian motion. Values >1 indicate a stronger signal than expected (although Λ is not always defined for values above 1) (Freckleton et al., 2002). Both phylogenetic signal metrics were calculated using the phylosig function from the *phytools* package (Revell, 2024).

### Root phenotypic networks and mycorrhizal associations

To assess the contribution of mycorrhizal fungi associations to root ecological strategies, we constructed and compared two root phenotypic networks: one including all measured traits (nine traits) and the other excluding colM (eight traits). In these networks, each trait included was represented as a node, and the phylogenetic correlations between trait pairs were considered as edges. We estimated correlations for each network using a phylogenetic variance-covariance matrix calculated with the phyl.vcv function from the *phytools* package (Revell, 2024). This phylogenetic matrix was then scaled into the corresponding Pearson pairwise correlation matrix of root traits using the cov2cor function from the *stats* base package of R. Only significant trait correlations (|r| > 0.5 and p < 0.05) were considered as trait connections in the network. The strength of meaningful trait-to-trait relationships was quantified using the absolute value of the resulting Pearson correlation coefficients (|r|). Correlations below the 0.5 threshold were set to zero, resulting in a weighted, undirected adjacency matrix A = [a_i,j_] with a_i,j_ ∈ [0,1].

We visualized and analyzed the correlation matrices (with and without colM) using the *igraph* package (Csárdi et al., 2024). For each weighted network, we calculated single-trait and whole-network parameters. To identify hub-traits, we calculated the weighted degree, of each trait using the strength R-function; traits with the highest weighted degrees were considered root hub-traits. We also assessed the strength of each trait’s connections (degree, k) and their centrality in the network (betweenness centrality) using the degree and betweenness functions in R. To determine whether the inclusion of colM significantly influenced these network parameters, we conducted paired t-tests, comparing parameter values for each root trait between the networks with and without colM (omitting colM).

We estimated overall trait independence in the networks as the average path length using the average.path.length function and measured network connectivity as edge density using the graph.density function. To evaluate overall trait phenotypic plasticity, we calculated the average clustering coefficient o using the transitivity function. Network integration was measured as connectance (C); that is, the fraction of links present in the network out of all possible links.

## Results

### Root trait covariation and mycorrhizal colonization

**Figure 1:**
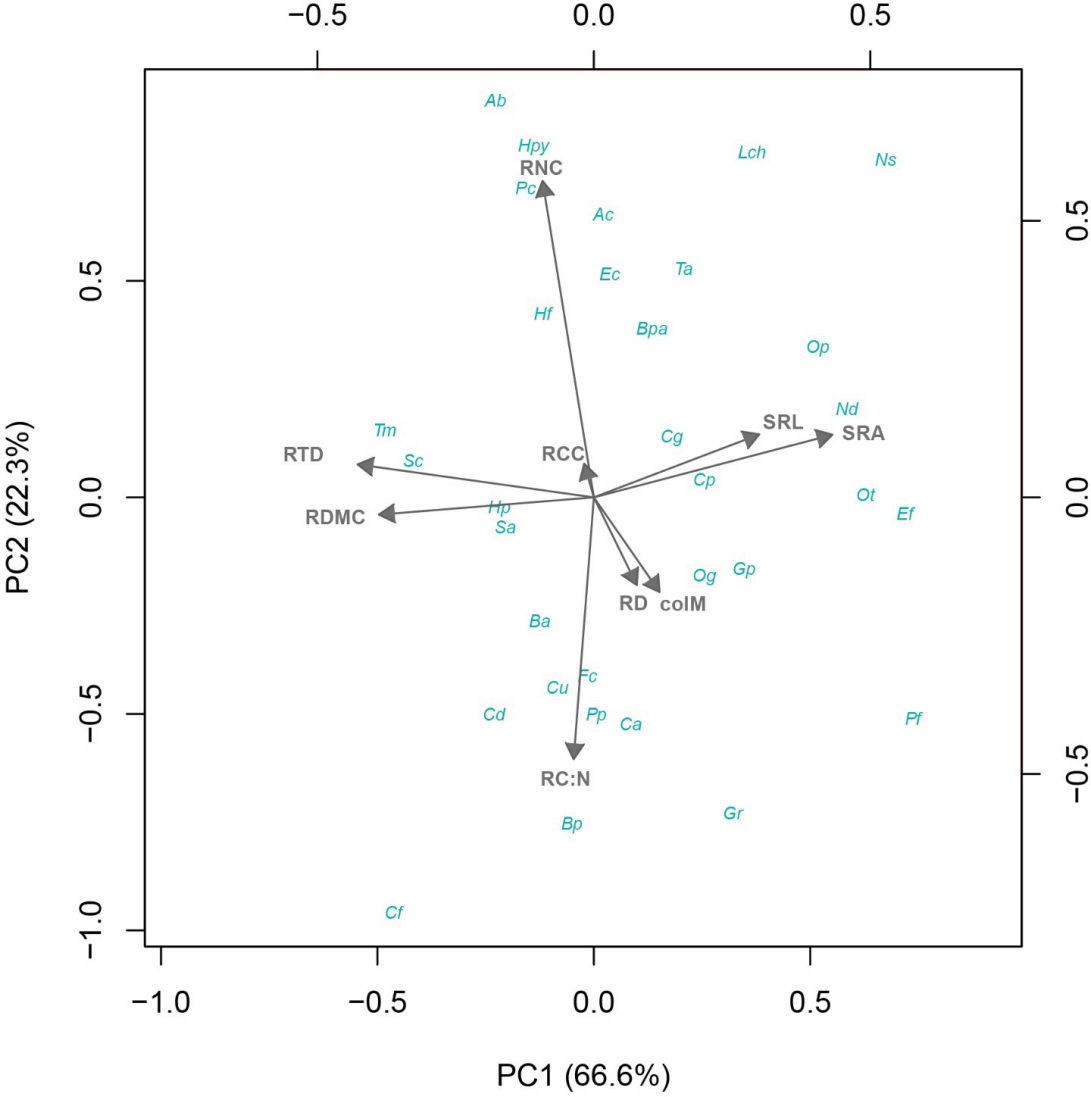
Phylogenetic principal component analysis of fine root traits from 32 shrub species native to the Coastal Atacama Desert. RD, root diameter; RTD, root tissue density; SRA, specific root area; SRL, specific root length; RDMC, root dry matter content; RNC, root N content; RC:N, root C:N ratio; RCC, root C content; colM, AM colonization.

**Figure 2:**
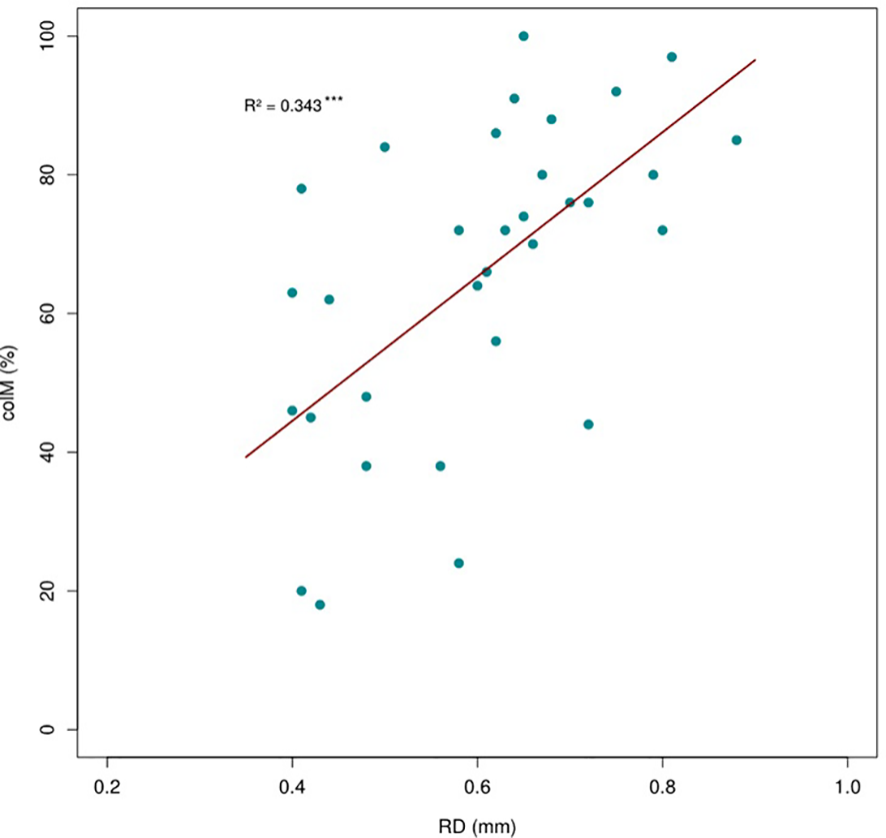
Positive relationship between root diameter (RD) and arbuscular mycorrhizal (AM) colonization across 32 shrub species. The regression line is based on a phylogenetic generalized linear model.

Our study revealed considerable variation in root traits among the shrub species studied. All species, including those in the order Caryophyllales, exhibited some degree of AM colonization (**Supplementary Table S2**; **Supplementary Figure S1**), although the percentage of AM colonization varied among species. In particular, species with larger RD tended to show higher levels of colM (**Figures 1**, **2**). We found significant correlations between colM and five other root traits (**Table 1**). Specifically, colM was positively associated with SRA and negatively associated with RTD, RDMC, RNC, and RCC. There was no significant association between colM and SRL. RD exhibited a strong negative correlation with RTD, RDMC, RNC, and RCC and a positive correlation with SRA. However, there was no clear relationship between RD and SRL. Additionally, RTD had a strong positive association with RDMC, but was negatively associated to SRL.

**Table 1:** Matrix of the Pearson’s phylogenetic correlation coefficients showing pairwise associations between eight root traits and AM colonization.

	RD	RTD	SRA	SRL	RDMC	RNC	RC:N	RCC
**colM**	**0.76**	**-0.68**	**0.52**	0.17	**-0.53**	**-0.56**	0.18	**-0.61**
**RD**		**-0.68**	**0.42**	-0.02	**-0.56**	**-0.52**	0.12	**-0.65**
**RTD**			**-0.90**	**-0.57**	**0.92**	0.27	0.13	**0.37**
**SRA**				**0.81**	**-0.88**	-0.09	-0.28	-0.22
**SRL**					**-0.63**	-0.04	-0.14	-0.08
**RDMC**						0.11	0.21	0.24
**RNC**							**-0.81**	**0.65**
**RC: N**								-0.23

Significant correlations at α = 0.05 are shown in bold. RD, root diameter; RTD, root tissue density; SRA, specific root area; SRL, specific root length; RDMC, root dry matter content; RNC, root N content; RC:N, root C:N ratio; RCC, root C content; colM, AM colonization.

The pPCA revealed a multidimensional trait gradient of variation across shrub species, showing significant trade-offs among root traits and colM in relation to resource acquisition and distribution (**Figure 1**). The first pPCA axis accounted for 66.6% of the total variation, representing a gradient driven mainly by morphological changes, characterized by the trade-off between SRL/SRA one side and RTD/RDMC on the other. The second axis, accounted for another 22.3% of the total variation, mainly capturing the trade-off between chemical traits, specifically RNC vs. RC:N. Both RD and colM exhibited similar patterns of variation across these two pPCA axes, highlighting their critical role in shaping root acquisition and survival strategies along the functional root gradient (**Table 2**; **Figure 1**).

**Table 2:** Results of the phylogenetic principal component analysis (pPCA) for all species.

	PC1	PC2
Standard deviation	0.228	0.132
Proportion of Variance	0.666	0.223
Cumulative Proportion	0.666	0.889
Trait	PC1	PC2
RD	0.435	-0.513
RTD	-0.974	0.079
SRA	0.974	0.148
SRL	0.836	0.184
RDMC	-0.963	-0.045
RNC	-0.267	0.950
RC:N	-0.126	-0.942
RCC	-0.286	0.574
colM	0.601	-0.500

RD, root diameter; RTD, root tissue density; SRA, specific root area; SRL, specific root length; RDMC, root dry matter content; RNC, root N content; RC:N, root C:N ratio; RCC, root C content; colM, AM colonization.

### Trait phylogenetic signal

We found that phylogenetic relatedness predicts the similarity of species in four root traits: RD, RTD, RCC, and RC:N (**Supplementary Table S3**). However, the phylogenetic signal for these traits was relatively low according to Blomberg’s K (between 0.13 and 0.21) and moderate according to Pagel’s Λ (between 0.47 and 0.77). This indicates that, while some phylogenetic influence exists, adaptive divergence may play a significant role in shaping these traits.

### Root phenotypic networks and mycorrhizal associations

**Figure 3:**
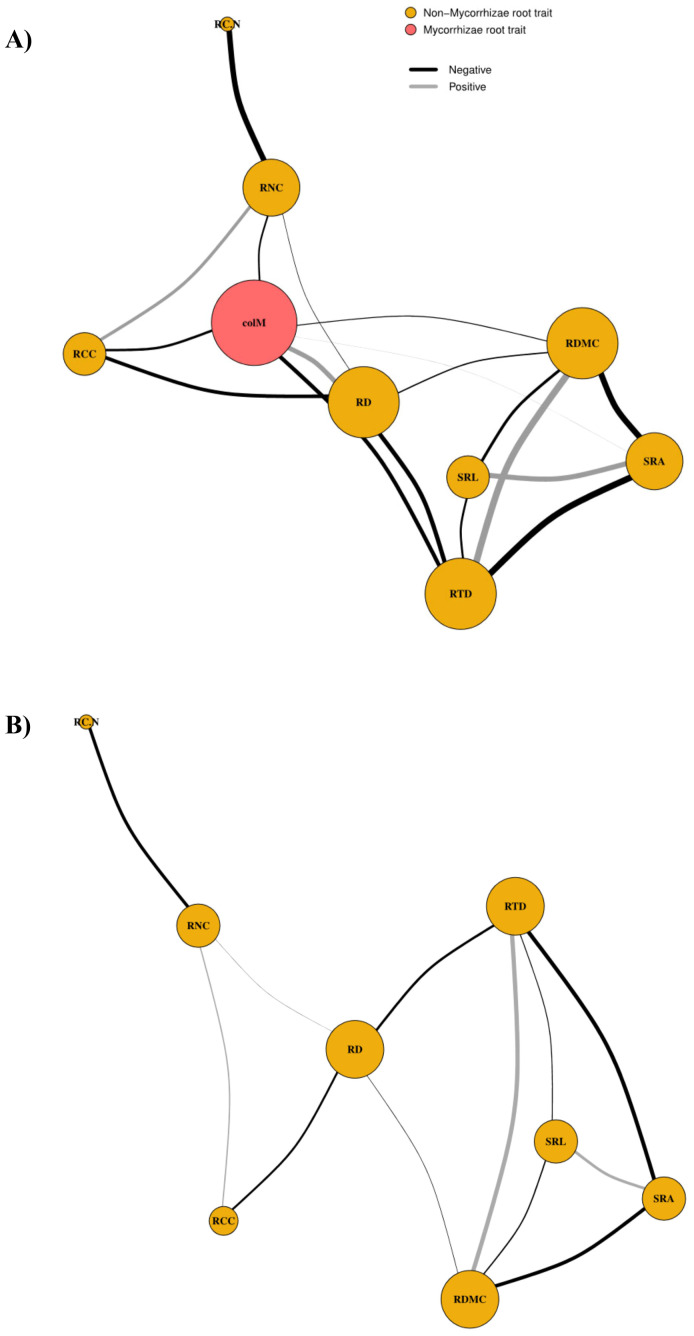
Root phenotypic networks with **(A)** mycorrhizal colonization (colM) included and **(B)** excluded from the network. Node size reflects betweenness centrality, indicating the importance of each trait in connecting the network. Links between the nodes represent significant positive (gray) or negative (black) trait correlations, with line width proportional to the strength of the correlation. RD, root diameter; RTD, root tissue density; SRA, specific root area; SRL, specific root length; RDMC, root dry matter content; RNC, root N content; RC:N, root C:N ratio; RCC, root C content; colM, AM colonization.

Network analysis revealed that excluding colM significantly alters the topological structure and single-trait connectivity of the root trait network (**Figures 3A, B**). The analysis shows a complex interplay between morphological and chemical root traits, with colM playing a central role in these relationships (**Table 3**). When included in the network, colM primarily forms negative associations with other traits (trade-offs), suggesting that it acts as a mediator of constraints within the overall functional root phenotype. Additionally, colM emerged as a hub trait, exhibiting some of the highest values in k, D, and betweenness centrality compared to the other traits (**Table 3**). When colM was removed from the network, there was a significant decrease in k and D values across all remaining traits (k: t = 4.58, df = 7, p = 0.003; D: t = 4.28, df = 7, p = 0.004). However, trait centrality in connecting the functional root phenotype was maintained (betweenness centrality: t = -1.43, df = 7, p = 0.197). Without colM, RD, RTD, RDMC, and SRA emerged as hub-traits, indicated by their D values (**Table 3**). When colM was included, the correlations of RD with other traits were strengthened; when colM was excluded, RD’s centrality diminished, though it still maintained significant correlations with other traits. This suggests that colM not only depends on root size but also modifies the role of RD in the network.

**Table 3:** Root trait network parameters and their ecological meaning (with and without Mycorrhizae colonization included in the network).

Parameter	Ecological meaning	Mycorrhizae	colM	RD	RTD	SRA	SRL	RDMC	RNC	RC:N	RCC
Single-trait network parameter											
Degree (k)	Strength of a trait connection to other traits	*Included*	6	5	5	4	3	5	4	1	3
		*Not included*	–	4	4	3	3	4	3	1	2
Weighted degree (D)	Indicator of a hub trait (most connected traits/important to the whole phenotype)	*Included*	3.7	3.2	3.8	3.1	2	3.5	2.5	0.8	1.9
		*Not included*	–	2.4	3.1	2.6	2	3	2	0.8	1.3
Betweenness centrality	Indicator of how central a trait is in connecting the phenotype	*Included*	0.3	0.2	0.0	0.0	0.0	0.2	0.3	0.0	0.0
		*Not included*	–	0.6	0.0	0	0	0.4	0.3	0.0	0.0
*Whole network parameter*			*Value*								
Average path length	Indicator of independence of traits	*Included*	1.1								
		*Not included*	1.3								
Edge density	Connectivity among traits for resource acquisition and distribution	*Included*	0.5								
		*Not included*	0.4								
Average clustering coefficient	Overall trait phenotypic plasticity to cope with environmental change	*Included*	0.7								
		*Not included*	0.6								
Connectance (C)	Overall phenotypic integration (whole plant efficiency in resource acquisition and distribution)	*Included*	0.4								
		*Not included*	0.3								

RD, root diameter; RTD, root tissue density; SRA, specific root area; SRL, specific root length; RDMC, root dry matter content; RNC, root N content; RC:N, root C:N ratio; RCC, root C content; colM, AM colonization.

At the whole-network level, removing colM increased trait independence, as indicated by a longer average path length among traits (**Table 3**). Additionally, the connectivity among traits related to resource acquisition and distribution, measured by edge density, decreased by 20%. Furthermore, the average clustering coefficient, which reflects the overall phenotypic plasticity of the traits, and C (overall phenotypic integration) decreased by ~15% and 25%, respectively, when colM is not present in the network.

## Discussion

Our study explored the intricate relationship between root traits and arbuscular mycorrhizal (AM) colonization in shrub species, revealing valuable insights into plant adaptation strategies in extremely arid ecosystems. Despite substantial variation in root trait attributes and the inclusion from species studied of different orders, including Caryophyllales, we consistently observed AM colonization. Notably, species with larger root diameters tended to exhibit higher levels of AM colonization, supporting the existence of an ‘outsourcing’ strategy along| the ‘collaboration gradient’. Furthermore, the significant correlations between AM colonization and other root traits highlight the complexity of the root phenotypic network, where AM colonization plays a central role in establishing connections among traits. These findings emphasize the importance of AM colonization in shaping root ecological strategies for shrub species in extreme arid ecosystems.

The results revealing that species with greater RD tend to have higher colonization rates support our hypothesis that fine root trait variation at the species level is linked with mycorrhizal fungi colonization, consistent with the ‘collaboration gradient’ (Bergmann et al., 2020). These findings suggest that thicker roots have a higher potential for colonization, likely because they provide more internal space for AM fungi. As RD increases, the cortex area expands faster than the stele, offering more habitat for fungi (Kong et al., 2019; Zhang et al., 2024). The negative relationship between RD and RTD further supports this, as parenchymatous cortical tissue is less dense than stele tissue (Valverde-Barrantes et al., 2016). A higher colM may enhance nutrient absorption in thicker roots, as AM are well-adapted to acquire N, P, and water from the soil (McCormack and Iversen, 2019; Freschet et al., 2021a). The phylogenetic conservatism observed in RD and RTD (**Supplementary Table S3**, **Supplementary Material**) suggests that this trait combination reflects an early-derived ecological strategy that enhances nutrient-foraging (mainly P) by maximizing cortex area available for AM colonization (Chen et al., 2013; Kong et al., 2017). In addition, our results show that colM and SRL are unrelated, contrasting the expected pattern of the ‘collaboration gradient’ expected in the RES. This may be explained by the fact that all species in our study have high RD values (> 0.4 mm, **Supplementary Table S2**, **Supplementary Material**), where sufficient cortex space exists for AM colonization regardless of SRL (Kong et al., 2014).

Although our results highlight the multidimensional nature of root traits, their patterns of covariation partially deviate from the expectations of the global RES. Specifically, our analysis shows that the second axis of the pPCA primarily captures the covariation between RNC and RC:N rather than with RTD. Interestingly, RD and colM are also associated with this second axis. Thus, species with higher RNC, lower RC:N, and thinner roots align with the ‘fast’ end of the spectrum, while species with lower RNC, high RC:N, and thicker roots occupy the ‘slow’ end. Fine root lifespan increases with RC:N and decreases with rising RNC (Hou et al., 2024), suggesting that the positive relationship between RC:N with root lifespan may attributed to higher concentrations of recalcitrant compounds, such as lignin and suberin, which enhance root resistance to herbivores and pathogens in the soil (Wardle et al., 2004).

Several studies have found a negative relationship between RNC and root lifespan (Withington et al., 2006; Reich, 2014; Hou et al., 2024), as RNC is often positively associated with higher metabolic activity and respiration rates (Reich et al., 2008; Roumet et al., 2016). However, RNC also plays other functional roles, such as facilitating nutrient uptake, enzyme activity, and N storage (Reich et al., 2008; Liu et al., 2015; Freschet et al., 2021a). In our study, RNC did not exhibit a significant phylogenetic signal (**Supplementary Table S3**, **Supplementary Material**), suggesting that its variation across species is likely shaped by environmental factors, such as soil fertility (Craine et al., 2005; Holdaway et al., 2011). Accordingly, a study of shrub communities in the southern CAD found a positive relationship between RNC and N soil availability (Delpiano et al., 2020), indicating that RNC is primarily influenced mainly by soil N availability in nutrient-limited environments (Zangaro et al., 2008; Zhang et al., 2018; Chapin et al., 1993). Although direct evidence linking RNC to root lifespan is limited, our findings suggest a complex interplay between RNC, environmental conditions, and root functional traits in arid ecosystems.

Several studies have consistently shown that RD plays a more significant role than RTD in determining root lifespan (McCormack et al., 2012; Ma et al., 2018; Kong et al., 2019; Hou et al., 2024), a finding that aligns with our results. McCormack et al. (2012) suggest that this relationship reflects a resource optimization strategy, where the increase in root lifespan with greater RD ensures better nutrient and water returns by investing more carbon in thicker roots than thinner ones. Additionally, higher colM in thicker roots may contribute to increased root lifespan due to the fungi’s role in plant defense against pathogens by producing secondary metabolites (Hou et al., 2024). Increased colM can also reduce root turnover rates, maximizing root foraging activity by expanding surface area (Aguilera et al., 2016; Shen et al., 2023) and enhancing resource availability (Augé, 2001; Allen, 2007; Qiao et al., 2019).

The phenotypic network analysis revealed that colM acts as a hub trait, highlighting its central role in structuring the root phenotypic network (He et al., 2020). In the hyper-arid CAD, colM mediates more functional trade-offs within the root phenotype than would be expected by chance (Bergmann et al., 2020). When colM is excluded from the network, traits such as RD, RTD, and RDMC become more prominent, but overall network connectivity declines. This underscores the role of colM in maintaining the interconnectedness of the root phenotypic network (Ye et al., 2024). These findings suggest that colM contributes to multiple root functions and shapes root ecological strategies (Freschet et al., 2021a).

The environmental conditions of the CAD impose several constraints on the survival of nearly all organisms (Maestre et al., 2015; Berdugo et al., 2020). Hyperaridity significantly limits water availability and slows biogeochemical cycles, resulting in low availability of soil nutrients like N, P, and K (Noy-Meir, 1973; Schlesinger et al., 1996). Given that shrubs in this region are long-lived, every individual will likely endure at least one period of intense drought during its lifespan (Carvajal et al., 2019). As a result, species must develop belowground strategies that extend root lifespan and optimize resource uptake when nutrients and water become available. In this context, having thick roots combined with high levels of colM seems to be the optimal functional strategy for shrubs to thrive under the extreme, hyper-arid conditions of the CAD.

## Conclusion

Our results offer new insights into plant adaptations in extreme arid ecosystems by linking root traits and arbuscular mycorrhizal colonization in 32 shrub species. We found that root trait covariation is multi-dimensional, with RD and colM playing key roles in shaping both dimensions of root ecological strategies. This finding differs from the global RES but aligns with recent studies linking RD and colM to the fast-slow ecological spectrum. To enhance our understanding of global root strategies, it is crucial to incorporate more quantitative data on root traits and AM colonization from underrepresented biomes. Network theory also proves to be a valuable complement PCA, effectively unraveling the complex, multidimensional relationships among root traits and their ecological roles. Given the significant role of AM in extreme environments, further research is needed to elucidate the mechanisms by which AM shapes root ecological strategies. In this context, the Coastal Atacama Desert provides an ideal natural laboratory for studying the effects of aridity on terrestrial ecosystems.

## Data Availability

The original contributions presented in the study are included in the article/**Supplementary Material**. Further inquiries can be directed to the corresponding author.
