# Supplementary material for: Arbuscular mycorrhizal colonization defines root ecological strategies in an extreme arid environment
Source: Front Plant Sci. 2025 Jan 15;15:1488383. doi: 10.3389/fpls.2024.1488383 (PMC11774874; doi:10.3389/fpls.2024.1488383)

# Arbuscular Mycorrhizal Colonization Defines Root Ecological Strategies in an Extreme Arid Environment

**Cristian A. Delpiano[1,2,3], Rodrigo S. Rios[1,2,3], Claudia E. Barraza-Zepeda[3], Melissa J. Pozo[3], Lorgio E. Aguilera[3], Andrea P. Loayza[1,2,3].**

[1]Laboratorio de Ecología del Desierto, Departamento de Biología, Universidad de La Serena, La Serena, Chile

[2]Instituto de Ecología y Biodiversidad (IEB), Santiago, Chile

[3]Departamento de Biología, Universidad de La Serena, La Serena, Chile

Correspondence:

Corresponding author e-mail: cdelpiano@userena.cl

**Table S1.** Details of the location and climatic characteristics of the six study sites. The De Martonne aridity index (DEMAI) was calculated as MAP/ (MAT + 10); thus, a lower DEMAI value indicates higher aridity. The climate data (mean annual precipitation, MAP; mean annual temperature, MAT) were sourced from Dirección General de Aguas, Chile (http://snia.dga.cl/BNAConsultas/reportes) and from CEAZA-Met weather station (www.ceazamet.cl)

| Site | Acronym | Coordinates | MAP (mm) | MAT (ºC) | DEMAI |
|---|---|---|---|---|---|
| Pan de Azúcar | **PAN** | 26º08'S, 70º39'W | 4 | 22.9 | 0.1 |
| Quebrada El León | **QL** | 26º57'S, 70º45'W | 14 | 17.2 | 0.5 |
| Llanos de Challe | **LLA** | 27º59'S, 71º07'W | 28 | 16.6 | 1.1 |
| Chañaral de Aceituno | **CHA** | 29º06'S, 71º27'W | 45 | 16.1 | 1.7 |
| Porotitos | **PO** | 29º43'S, 71º19'W | 80 | 15.3 | 3.2 |
| Fray Jorge | **FJ** | 30º38'S, 71º40'W | 147 | 13.6 | 6.2 |

**Table S2.** Mean and standard error of the mean (SE) for arbuscular mycorrhizal colonization and root traits measured in 32 shrub species in the coastal Atacama Desert (Chile). RD, root diameter; RTD, root tissue density; SRA, specific root area; SRL, specific root length; RDMC, root dry matter content; RNC, root N content; RC:N, root C:N ratio; RCC, root C content; colM: AM colonization. FJ, Fray Jorge; PO, Porotitos; CHA, Chañaral de Aceituno; LLA, Llanos de Challe; QL, Quebrada El León; PAN, Pan de Azúcar.

| Order | Family | Species | Site | colM (%) | RD (mm) | SRL (m gr$^{-1}$) | SRA (cm$^2$ gr$^{-1}$) | RTD (g cm$^{-3}$) | RDMC (mg gr$^{-1}$) | RNC (%) | RCC (%) | RC:N |
|---|---|---|---|---|---|---|---|---|---|---|---|---|
| Asterales | Asteraceae | *Baccharis paniculata* DC. | FJ - PO | 76 ± 8 | 0.70 ± 0.05 | 6.33 ± 0.83 | 122.75 ± 9.10 | 0.52 ± 0.04 | 447.32 ± 24.16 | 1.53 ± 0.08 | 39.75 ± 1.15 | 26.61 ± 1.28 |
| Asterales | Asteraceae | *Bahia ambrosioides* Lag. | PO | 80 ± 10 | 0.67 ± 0.09 | 5.54 ± 1.18 | 104.91 ± 15.44 | 0.65 ± 0.11 | 483.5 ± 68.36 | 0.93 ± 0.04 | 40.60 ± 1.70 | 43.75 ± 1.66 |
| Asterales | Asteraceae | *Chuquiraga ulicina* (Hook. & Arn.) Hook. & Arn. | CHA - QL | 66 ± 10 | 0.61 ± 0.05 | 6.38 ± 0.92 | 111.74 ± 12.47 | 0.66 ± 0.05 | 493.03 ± 22.80 | 0.83 ± 0.06 | 45.42 ± 3.91 | 56.77 ± 7.55 |
| Asterales | Asteraceae | *Encelia canescens* Lam. | CHA – LLA - QL | 86 ± 6 | 0.62 ± 0.03 | 6.49 ± 1.11 | 167.79 ± 45.42 | 0.75 ± 0.11 | 494.63 ± 36.21 | 1.27 ± 0.17 | 44.95 ± 1.13 | 41.64 ± 4.68 |
| Asterales | Asteraceae | *Erigeron fasciculatus* Colla | PO | 100 ± 0 | 0.65 ± 0.06 | 8.14 ± 1.47 | 158.04 ± 14.88 | 0.41 ± 0.03 | 311.44 ± 12.70 | 1.17 ± 0.28 | 42.91 ± 1.04 | 40.98 ± 9.21 |
| Asterales | Asteraceae | *Gutierrezia resinosa* (Hook. & Arn.) S.F. Blake | FJ – PO | 91 ± 4 | 0.64 ± 0.04 | 7.22 ± 1.19 | 125.50 ± 16.46 | 0.50 ± 0.03 | 409.93 ± 24.66 | 0.67 ± 0.06 | 42.56 ± 0.89 | 66.61 ± 6.03 |
| Asterales | Asteraceae | *Gypothamnium pinifolium* Phil. | PAN | 72 ± 17 | 0.63 ± 0.03 | 8.36 ± 1.17 | 162.88 ± 20.59 | 0.42 ± 0.05 | 629.51 ± 23.94 | 0.98 ± 0.09 | 41.64 ± 6.04 | 45.53 ± 9.87 |
| Asterales | Asteraceae | *Haplopappus parvifolius* (DC.) Gay | PO | 76 ± 15 | 0.72 ± 0.07 | 4.55 ± 0.46 | 97.79 ± 6.13 | 0.60 ± 0.06 | 485.31 ± 30.23 | 1.21 ± 0.10 | 41.86 ± 0.16 | 35.52 ± 2.97 |

| | | | | | | | | | | | | |
|---|---|---|---|---|---|---|---|---|---|---|---|---|
| Asterales | Asteraceae | *Ophryosporus paradoxus* (Hook. & Arn.) Benth. & Hook. ex B.D. Jacks. | PO | 44 ± 17 | 0.72 ± 0.06 | 6.49 ± 1.39 | 138.13 ± 17.59 | 0.43 ± 0.04 | 339.93 ± 21.26 | 1.42 ± 0.25 | 40.46 ± 0.43 | 30.69 ± 6.51 |
| Asterales | Asteraceae | *Ophryosporus triangularis* Meyen | LLA | 56 ± 9 | 0.62 ± 0.04 | 9.15 ± 2.21 | 169.63 ± 32.04 | 0.42 ± 0.05 | 294.23 ± 66.89 | 1.09 ± 0.26 | 42.37 ± 1.39 | 42.29 ± 7.38 |
| Asterales | Asteraceae | *Polyachyrus fuscus* (Meyen) Walp. | LLA - QL | 85 ± 5 | 0.88 ± 0.06 | 6.57 ± 1.81 | 153.45 ± 26.04 | 0.36 ± 0.03 | 294.45 ± 41.44 | 0.81 ± 0.07 | 42.23 ± 1.44 | 53.64 ± 4.54 |
| Asterales | Asteraceae | *Polyachyrus poeppigii* Kuntze ex Less. | PO | 72 ± 16 | 0.80 ± 0.11 | 5.13 ± 1.94 | 103.45 ± 14.81 | 0.54 ± 0.04 | 421.35 ± 33.50 | 0.89 ± 0.25 | 43.12 ± 0.94 | 56.18 ± 13.8 |
| Asterales | Asteraceae | *Proustia cuneifolia* D. Don | FJ | 38 ± 13 | 0.48 ± 0.06 | 9.44 ± 1.52 | 128.75 ± 14.66 | 0.70 ± 0.04 | 633.04 ± 36.16 | 1.83 ± 0.39 | 42.35 ± 1.99 | 27.6 ± 5.90 |
| Boraginales | Heliotropiaceae | *Heliotropium floridum* (A. DC.) Clos | LLA – QL | 78 ± 9 | 0.41 ± 0.03 | 10.64 ± 2.25 | 121.32 ± 17.04 | 0.90 ± 0.06 | 561.48 ± 29.07 | 1.14 ± 0.12 | 37.90 ± 1.64 | 36.5 ± 5.19 |
| Boraginales | Heliotropiaceae | *Heliotropium pycnophyllum* Phil. | PA | 62 ± 18 | 0.44 ± 0.08 | 11.56 ± 4.54 | 117.21 ± 25.04 | 0.94 ± 0.07 | 540.54 ± 37.77 | 1.77 ± 0.24 | 38.01 ± 1.53 | 22.88 ± 2.69 |
| Caryophyllales | Aizoaceae | *Tetragonia angustifolia* Barnéoud | QL - PA | 63 ± 13 | 0.40 ± 0.03 | 14.84 ± 3.02 | 158.49 ± 23.61 | 0.80 ± 0.08 | 563.71 ± 21.15 | 1.25 ± 0.10 | 32.10 ± 1.06 | 27.29 ± 2.56 |
| Caryophyllales | Aizoaceae | *Tetragonia maritima* Barnéoud | CHA | 72 ± 15 | 0.58 ± 0.04 | 5.39 ± 0.83 | 95.64 ± 12.76 | 0.76 ± 0.07 | 699.74 ± 39.90 | 1.24 ± 0.13 | 35.33 ± 1.67 | 30.11 ± 3.92 |

| | | | | | | | | | | | | |
|---|---|---|---|---|---|---|---|---|---|---|---|---|
| Caryophyllales | Chenopodiaceae | *Atriplex clivicola* I.M. Johnst. | LLA | 20 ± 12 | 0.41 ± 0.03 | 12.66 ± 2.88 | 153.39 ± 29.51 | 0.81 ± 0.19 | 596.57 ± 43.40 | 1.41 ± 0.08 | 39.5 ± 1.31 | 28.44 ± 1.99 |
| Caryophyllales | Chenopodiaceae | *Chenopodium petiolare* Kunth | FJ | 48 ± 7 | 0.48 ± 0.07 | 10.58 ± 2.37 | 143.55 ± 24.20 | 0.65 ± 0.06 | 448.97 ± 22.43 | 1.00 ± 0.24 | 38.4 ± 2.38 | 42.41 ± 8.97 |
| Caryophyllales | Frankeniaceae | *Frankenia chilensis* K. Presl | LLA | 46 ± 9 | 0.40 ± 0.02 | 10.10 ± 1.91 | 126.08 ± 26.47 | 0.93 ± 0.16 | 450.33 ± 38.68 | 0.66 ± 0.08 | 36.55 ± 6.46 | 58.84 ± 15.05 |
| Caryophyllales | Polygonaceae | *Chorizanthe desertícola* Phil. | CHA | 18 ± 7 | 0.43 ± 0.06 | 11.03 ± 3.93 | 122.49 ± 29.87 | 0.92 ± 0.10 | 657.60 ± 28.47 | 0.66 ± 0.06 | 47.75 ± 0.62 | 74.96 ± 6.61 |
| Caryophyllales | Polygonaceae | *Chorizanthe frankenioides* J. Remy | PO | 92 ± 4 | 0.75 ± 0.12 | 4.24 ± 1.57 | 78.42 ± 9.17 | 0.75 ± 0.08 | 535.74 ± 71.92 | 0.61 ± 0.03 | 40.72 ± 1.09 | 66.85 ± 3.53 |
| Fabales | Fabaceae | *Adesmia bedwellii* Skottsb . | FJ | 24 ± 16 | 0.58 ± 0.06 | 6.88 ± 1.28 | 111.69 ± 9.66 | 0.64 ± 0.02 | 555.79 ± 38.54 | 2.22 ± 0.38 | 42.20 ± 1.87 | 21.67 ± 4.32 |
| Fabales | Fabaceae | *Senna cumingii* (Hook. & Arn.) H.S. Irwin & Barneby | PO | 80 ± 15 | 0.79 ± 0.04 | 3.97 ± 0.53 | 93.86 ± 10.30 | 0.59 ± 0.05 | 638.79 ± 41.63 | 1.44 ± 0.24 | 42.02 ± 3.27 | 32.94 ± 5.91 |
| Gentianales | Apocynaceae | *Skytanthus acutus* Meyen | LLA – QL - PAN | 64 ± 9 | 0.60 ± 0.03 | 6.52 ± 0.95 | 111.54 ± 11.3 | 0.77 ± 0.1 | 528.29 ± 35.12 | 1.04 ± 0.07 | 39.29 ± 2.18 | 40.39 ± 4.56 |
| Geraniales | Francoaceae | *Balbisia peduncularis* (Lindl.) D. Don | LLA | 84 ± 8 | 0.50 ± 0.02 | 7.90 ± 0.81 | 119.57 ± 7.65 | 0.69 ± 0.03 | 588.84 ± 19.81 | 0.62 ± 0.05 | 43.72 ± 0.53 | 72.74 ± 5.89 |
| Malvales | Malvaceae | *Cristaria aspera* Gay | QL | 97 ± 2 | 0.81 ± 0.07 | 5.03 ± 1.33 | 103.42 ± 16.51 | 0.55 ± 0.03 | 390.69 ± 21.76 | 0.82 ± 0.07 | 37.41 ± 0.92 | 47.23 ± 3.64 |
| Malvales | Malvaceae | *Cristaria glaucophylla* | FJ - PO | 88 ± 12 | 0.68 ± 0.04 | 5.88 ± 1.22 | 124.59 ± 25.67 | 0.56 ± 0.11 | 372.51 ± 50.24 | 1.32 ± 0.24 | 40.60 ± 0.89 | 33.26 ± 7.35 |

| | | | | | | | | | | | | |
|---|---|---|---|---|---|---|---|---|---|---|---|---|
| | | Cav. | | | | | | | | | | |
| Oxalidales | Oxalidaceae | *Oxalis gigantea* Barnéoud | PO - LLA | 45 ± 15 | 0.42 ± 0.02 | 11.51 ± 1.22 | 144.96 ± 15.68 | 0.72 ± 0.08 | 409.34 ± 29.2 | 0.86 ± 0.08 | 47.85 ± 0.81 | 58.14 ± 5.65 |
| Solanales | Solanaceae | *Lycium chilense* Miers ex Bertero | FJ | 70 ± 14 | 0.66 ± 0.09 | 8.18 ± 2.64 | 146.68 ± 29.69 | 0.46 ± 0.04 | 438.84 ± 46.59 | 2.05 ± 0.27 | 43.8 ± 0.89 | 21.94 ± 2.37 |
| Solanales | Solanaceae | *Nolana divaricata* (Lindl.) I.M. Johnst. | CHA | 74 ± 15 | 0.65 ± 0.09 | 10.93 ± 3.6 | 180.43 ± 42.61 | 0.44 ± 0.06 | 500.50 ± 77.56 | 1.19 ± 0.11 | 38.04 ± 1.92 | 33.48 ± 4.27 |
| Solanales | Solanaceae | *Nolana sedifolia* Poepp. | CHA | 38 ± 16 | 0.56 ± 0.05 | 11.08 ± 2.18 | 182.43 ± 20.04 | 0.41 ± 0.03 | 387.33 ± 67.15 | 1.74 ± 0.17 | 40.66 ± 1.04 | 23.87 ± 2.75 |

**Table S3.** Phylogenetic signals Blomberg's *K* and Pagel λ of the eight root traits, Arbuscular mycorrhizal colonization for all species, and the two phylogenetical principal component axes. Bold is indicative of a significant phylogenetic signal. RD, root diameter; RTD, root tissue density; SRA, specific root area; SRL, specific root length; RDMC, root dry matter content; RNC, root N content; RC:N, root C:N ratio; RCC, root C content; colM: AM colonization.

| **Trait** | **Blomberg's *K*** | **P-value** | **Pagel's λ** | **P-value** |
|---|---|---|---|---|
| **RD** | 0.13 | 0.079 | 0.47 | **0.035** |
| **SRL** | 0.15 | 0.062 | 0.29 | 0.158 |
| **SRA** | 0.03 | 0.752 | 0.10 | 0.779 |
| **RTD** | 0.09 | 0.212 | 0.77 | **<0.001** |
| **RNC** | 0.13 | 0.078 | 0.32 | 0.399 |
| **RCC** | 0.21 | **0.021** | 0.73 | **0.005** |
| **RC:N** | 0.18 | **0.036** | 0.66 | **0.028** |
| **RDMC** | 0.06 | 0.365 | 0.27 | 0.223 |
| **colM** | 0.12 | 0.133 | 0.18 | 0.505 |
| **PC1** | 0.02 | 0.815 | 0.25 | 0.223 |
| **PC2** | 0.14 | 0.059 | 0.43 | 0.162 |

**Figure S1.** Microscopy images (Nikon Eclipse E200, 400× magnification) of arbuscular mycorrhizal colonization in six shrub species from the Coastal Atacama Desert. a) *Tetragonia angustifolia* (QL) b) *Atriplex clivicola* (LLA) c) *Chorizanthe deserticola* (PO), d) *Chenopodium petiolare* FJ e) *Heliotropium floridum* (LLA) and f) *Skythantus acutus* (PA).

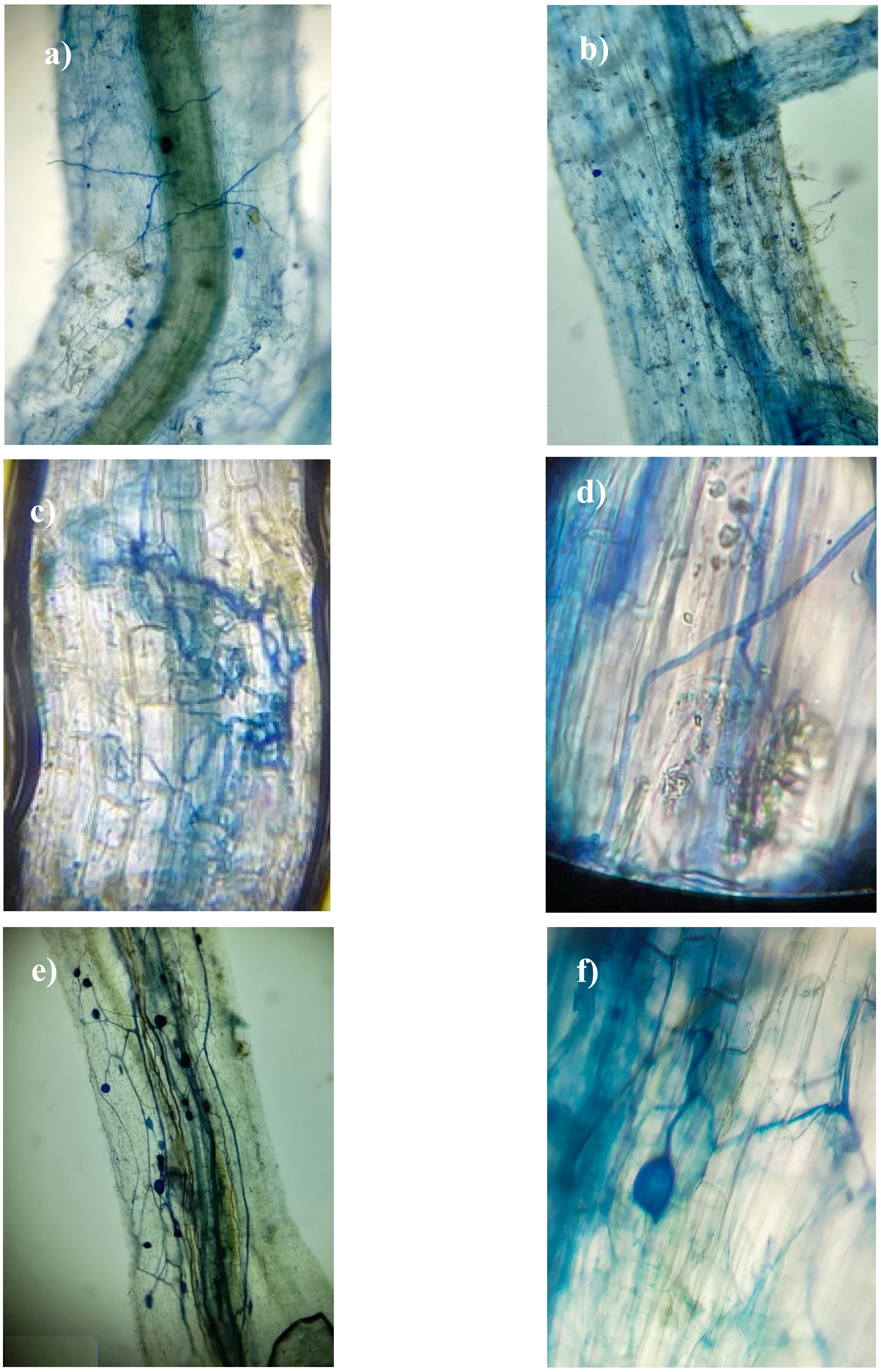

Supplement: Supplementary file 1 [file DataSheet1.pdf]
